# The EPIPHA-KNEE trial: Explaining Pain to target unhelpful pain beliefs to Increase PHysical Activity in KNEE osteoarthritis – a protocol for a multicentre, randomised controlled trial with clinical- and cost-effectiveness analysis

**DOI:** 10.1186/s12891-021-04561-6

**Published:** 2021-08-28

**Authors:** Tasha R. Stanton, Felicity A. Braithwaite, David Butler, G. Lorimer Moseley, Catherine Hill, Rachel Milte, Julie Ratcliffe, Carol Maher, Christy Tomkins-Lane, Brian W. Pulling, Erin MacIntyre, Adrian Esterman, Ty Stanford, Hopin Lee, Francois Fraysse, Ben Metcalf, Brendan Mouatt, Kim Bennell

**Affiliations:** 1 IIMPACT in Health, Allied Health and Human Performance, University of South Australia, G.P.O. Box 2471, Adelaide, 5001 Australia; 2 NOIgroup Pty Ltd, Adelaide, South Australia; 3 Rheumatology Department, The Queen Elizabeth Hospital, Adelaide, Australia; 4 Adelaide Medical School, Faculty of Health and Medical Sciences, The University of Adelaide, Adelaide, Australia; 5 Caring Futures Institute, Flinders University, Adelaide, Australia; 6 Alliance for Research in Exercise, Nutrition and Activity (ARENA), Allied Health and Human Performance, University of South Australia, Adelaide, Australia; 7 Department of Health and Physical Education, Mount Royal University, Calgary, Canada; 8 Clinical & Health Sciences, University of South Australia, Adelaide, Australia; 9 Centre for Statistics in Medicine, Rehabilitation Research in Oxford (RRIO), Nuffield Department of Orthopaedics, Rheumatology and Musculoskeletal Sciences (NDORMS), University of Oxford, Oxford, UK; 10 School of Medicine and Public Health, University of Newcastle, Newcastle, Australia; 11 Centre for Health, Exercise and Sports Medicine, Department of Physiotherapy, The University of Melbourne, Victoria, Australia

**Keywords:** Osteoarthritis, Physical activity, Exercise, Walking program, Strengthening program, Pain science education, RCT, Physiotherapy

## Abstract

**Background:**

Despite well-established benefits of physical activity for knee osteoarthritis (OA), nine of ten people with knee OA are inactive. People with knee OA who are inactive often believe that physical activity is dangerous, fearing that it will further damage their joint(s). Such unhelpful beliefs can negatively influence physical activity levels. We aim to evaluate the clinical- and cost-effectiveness of integrating physiotherapist-delivered pain science education (PSE), an evidence-based conceptual change intervention targeting unhelpful pain beliefs by increasing pain knowledge, with an individualised walking, strengthening, and general education program.

**Methods:**

Two-arm, parallel-design, multicentre randomised controlled trial involving 198 people aged ≥50 years with painful knee OA who do not meet physical activity guideline recommendations or walk regularly for exercise. Both groups receive an individualised physiotherapist-led walking, strengthening, and OA/activity education program via 4x weekly in-person treatment sessions, followed by 4 weeks of at-home activities (weekly check-in via telehealth), with follow-up sessions at 3 months (telehealth) and 5 and 9 months (in-person). The EPIPHA-KNEE group also receives contemporary PSE about OA/pain and activity, embedded into all aspects of the intervention. Outcomes are assessed at baseline, 12 weeks, 6 and 12 months. Primary outcomes are physical activity level (step count; wrist-based accelerometry) and self-reported knee symptoms (WOMAC Total score) at 12 months. Secondary outcomes are quality of life, pain intensity, global rating of change, self-efficacy, pain catastrophising, depression, anxiety, stress, fear of movement, knee awareness, OA/activity conceptualisation, and self-regulated learning ability. Additional measures include adherence, adverse events, blinding success, COVID-19 impact on activity, intention to exercise, treatment expectancy/perceived credibility, implicit movement/environmental bias, implicit motor imagery, two-point discrimination, and pain sensitivity to activity. Cost-utility analysis of the EPIPHA-KNEE intervention will be undertaken, in addition to evaluation of cost-effectiveness in the context of primary trial outcomes.

**Discussion:**

We will determine whether the integration of PSE into an individualised OA education, walking, and strengthening program is more effective than receiving the individualised program alone. Findings will inform the development and implementation of future delivery of PSE as part of best practice for people with knee OA.

**Trial registration:**

Australian New Zealand Clinical Trials Registry: ACTRN12620001041943 (13/10/2020).

**Supplementary Information:**

The online version contains supplementary material available at 10.1186/s12891-021-04561-6.

## Background

Osteoarthritis (OA) is increasingly prevalent in older adults [1] and has a large personal health and societal burden [2]. The knee is the most common joint affected by OA and is the most disabling [1], making it essential to find ways to promote long-term health, quality of life, and reduced pain and disability in this population.

Regular structured physical activity (aerobic or strengthening exercise) reduces pain and disability in people with knee OA [3, 4], even in those with severe, end-stage OA who are awaiting joint replacement [5]. Recommended by all clinical guidelines as a core treatment for knee OA, structured physical activity has similar effects on pain and disability as commonly used drugs, but without the pharmacological side effects (e.g., gastrointestinal problems) [3, 4]. Importantly, even relatively small improvements in physical activity levels predict improved function [6] and reduced disability [7] in those with knee OA. Despite this, the majority of people with OA are inactive [8]. Only 13% of people with OA meet physical activity guidelines for moderate-vigorous activity (150 min/week) and only 19% meet suggestions of ≥10,000 steps/day [8]. While most people in the general public do not meet recommended activity levels, people with OA are still 25% less active than those without OA [8]. Low physical activity levels also have serious health implications for people with knee OA: cardiovascular mortality risk in those with OA is nearly double that of the general population and is highest for those with the greatest walking disability [9].

Recent literature supports that unhelpful beliefs likely contribute to inactivity in those with knee OA. For example, in people with OA, those who are inactive are more likely than those who are active to believe that they are physically unable to exercise and that physical activity is unsafe [10, 11]. Further, people with knee OA often focus heavily on pain and hold beliefs that OA is an incurable, progressive ‘bone-on-bone’ disease, caused by ‘wear-and-tear’ [12, 13]; perspectives refuted by contemporary scientific evidence [14–18]. Given such beliefs, it is perhaps unsurprising that people with knee OA generally report increased levels of fear of movement itself and of movement-induced injury [19, 20] and report uncertainty as to whether undertaking physical activity will be helpful for them [21]. Unfortunately, these unhelpful beliefs are also held by many health care professionals, who reinforce consumers’ misunderstandings, further compounding the problem. For example, many clinicians believe that structured physical activity is not appropriate for all people with knee OA (i.e., not appropriate for people with severe OA) and that physical activity can be harmful [22]. Such consumer and clinician beliefs contrast with high-quality research showing that physical activity does not further damage the joint [3, 23], and that activity is helpful even in those with end-stage OA awaiting joint replacement [5]. Critically, in people with knee OA, such unhelpful beliefs negatively influence their decision to engage with structured physical activity [12] and reduce their participation in activities that may be pain-provoking [24]. Ultimately, negative beliefs about OA and activity are predictive of lower future physical activity levels in those with knee OA [25].

Current strategies that aim to increase physical activity in people with OA include structured physical activity interventions targeting aerobic capacity, endurance, and strength [26]. These interventions aim to increase exercise tolerance, strength or overall fitness, maintain or reduce weight, and decrease pain. They have short-term but not long-term effects on physical activity levels [26]. Behavioural interventions, targeting features such as self-efficacy or coping skills to promote self-management, have also been used to increase physical activity. Again, physical activity levels increase in the short term, but decline over time, with no effect after 12 months [27]. Critically, the reduction in benefits from physical activity in OA is directly related to declining rates of adherence [28]. Thus, improving long-term adherence is key to achieving meaningful health and OA-specific benefits. Promoting maintenance of physical activity gains remains a significant challenge – we are still chasing the holy grail of instilling physical activity as a regular part of daily life in people with OA.

A notable similarity amongst most current approaches that aim to increase physical activity is encouraging ‘movement despite pain’ (e.g., behavioural interventions using coping skills), with the ‘promise’ of pain-relieving effects over time. Arguably, such approaches may seem counterintuitive to people with knee OA as long as they consider pain to be a marker of joint damage [29]. For instance, if people believe that increases in pain represent more joint damage and that physical activity may be unsafe for their joint, then it is unlikely that they will engage in or adhere to activity programs that suggest that they should move despite having pain [12]. Further, if these unhelpful beliefs are not challenged, then flare-ups of pain that often occur with activity programs may derail long-term adherence [30]. Although including general education within physical activity programs is recommended by OA practice guidelines [31], we contend that current education may be suboptimal. Current education largely focuses on how to accurately and safely perform activity and the health and OA-specific benefits of activity [32]. Such education does not improve poor long-term activity adherence [33].

Contemporary pain science education (PSE) was developed to shift the meaning of pain from that of a marker of tissue damage to that of a need to protect the body from real or perceived danger or threat. PSE aims to re-conceptualise what pain *means* to someone [29]. It is distinct from cognitive therapy because it uses re-conceptualisation of the biological underpinnings of pain to enable a move away from thinking that only structural pathological factors contribute to pain. PSE also prioritises an understanding of the considerable central nervous system adaptations that occur when pain persists and the impact of these changes on the relationship between pain and true tissue threat. PSE therefore, provides a scientific basis for both a biopsychosocial model of pain and disability (i.e., underscoring that numerous factors can contribute to the experience of pain), and the enhanced sensitivity generated by central nervous system adaptations that occur as pain persists [29]. Importantly, PSE also differs fundamentally from current self-management education programs that aim to educate people with OA about physical activity benefits and behavioural modifications (and which have been shown to have no benefit in OA [34]), in that PSE targets the knowledge and beliefs that underlie behaviour [29]. With PSE, re-conceptualisation of pain is key: those with the greatest improvement in pain-related knowledge with PSE show the greatest immediate *and sustained* movement improvements [35].

High level evidence, including meta-analyses and randomised controlled trials (RCTs), shows that PSE increases pain knowledge, reduces unhelpful pain beliefs [35, 36], and improves pain, function, and disability (including physical activity levels) in various musculoskeletal pain conditions [35–41]. However, knee OA-specific data are lacking. We undertook a feasibility pilot study of a PSE-driven individualised education and walking program for people with knee OA [42]. The OA PSE intervention drew from established ‘Explain Pain’ programs (originally developed for back pain; see Moseley & Butler for an overview [29]), that were then modified to include contemporary understanding of the science of OA [16, 17, 43], including the impact of movement and loading of the knee joints [15, 18, 44]. The PSE intervention had high levels of participant-rated credibility and acceptability, with promising effects on pain knowledge and clinical outcomes of pain, function, and physical activity levels [42]. Our findings supported transition to a full trial after undertaking small methodological changes, and including a lower limb strengthening component to be consistent with best practice care [31].

Therefore, the aim of this study is to determine the clinical- and cost-effectiveness of a PSE-driven individualised walking, strengthening and education program, called EPIPHA-KNEE, in people with painful knee OA. We will compare EPIPHA-KNEE to an individualised walking, strengthening, and general OA educational program that is consistent with current best practice, using a randomised controlled trial (RCT). We hypothesise that the EPIPHA-KNEE intervention will lead to greater improvements in long-term physical activity levels and OA knee symptoms (pain, function, and stiffness) and will be cost-effective.

## Methods

### Design

**Fig. 1:**
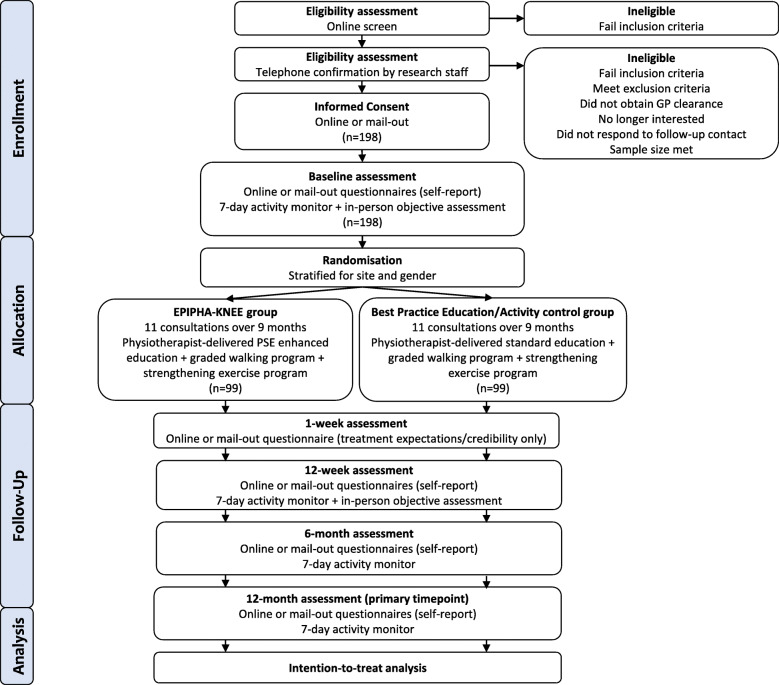
Participant flow through the randomised controlled trial

EPIPHA-KNEE is a two-arm, multicentre, superiority RCT. Figure 1 outlines the phases of the RCT. This protocol has been developed according to the SPIRIT recommendations [45]. The intervention is described according to the TIDieR checklist [46]. The trial was prospectively registered with the Australian New Zealand Clinical Trial Registry (ACTRN12620001041943; https://bit.ly/2SfVySS). The trial is conducted at the University of South Australia and the University of Melbourne, with cost-effectiveness analysis led by health economists at Flinders University, Australia.

### Participants

Participants aged ≥50 years with painful knee OA that has been present for at least 6 months, who have at least moderate levels of pain (≥4 on an 11-point Numerical Pain Rating Scale [47]) and who report at least moderate difficulty with daily activities [48] will be eligible for this trial. Table 1 provides the full inclusion and exclusion criteria. Our target sample size is 198 participants. Participants are recruited from the general community in Adelaide and Melbourne, Australia using advertisements placed in newspapers and relevant newsletters (e.g., Arthritis SA, Senior’s Living), as well as via social media (Facebook, Twitter).

**Table 1:** Participant eligibility criteria

Inclusion criteria	Exclusion criteria
Fulfil the National Institute for Health and Care Excellence (NICE) clinical criteria for osteoarthritis: • Age ≥ 50 years;^a^ • Have activity related joint pain; and • Have morning stiffness ≤30 min.	Health conditions that prevent safe participation in physical activity interventions as listed in the American College of Sports Medicine Guidelines (e.g., cardiac or lung disease)
Average knee pain intensity (overall and/or while walking) of ≥4 on 11-point numeric rating scale (NRS, where 0 = no pain; 10 = worst pain possible) in the past week	For those identified as at risk from the pre-exercise screening, general practitioner does not give medical clearance
Moderate disability due to the knee over the past week (≥ 3 on the Global Disability Rating Scale)	Pain in other body areas that currently limits walking ability (e.g., back pain, foot pain, hip pain)
Knee pain of at least 6 months duration	Neurological disorders affecting lower limb movement (e.g., multiple sclerosis or stroke)
Current levels of moderate/vigorous physical activity (MVPA) below physical activity guideline recommendations (< 150 min/week of MVPA; assessed using the Active Australia Physical Activity Questionnaire)	Inflammatory arthritis (including rheumatoid arthritis)
Current purposeful walking for exercise of ≤30 mins/day on ≤5 days/week	Fibromyalgia
Able to give informed consent and to participate fully in the interventions and the assessment procedures	Planned knee replacement/surgery (next 12 months); and/or recent knee replacement on the non- or less-painful knee (< 6 months)
	Previously operated knee is the most painful knee
	Intra-articular therapy use in the 12 weeks preceding enrolment
	Any condition impacting decision-making/memory (e.g., Alzheimer’s, dementia)
	Severe depression (> 17 on the 4-item PROMIS depression subscale)
	Current moderate/vigorous physical activity levels above guideline recommendations (≥150 min/week; assessed using the Active Australia Physical Activity Questionnaire)
	Current purposeful walking for exercise of ≥30 mins/day on ≥5 days/week.
	Currently undergoing regular, active intervention for the knee (e.g., seeing a physiotherapist)
	Unable to commit to study requirements (unable to attend study appointments or complete study outcomes)

^a^Note: NICE criteria for age is > 45 years; however, ≥ 50 years has been used for the present trial to limit inclusion of younger participants with trauma-induced OA

Trial coordinators at the University of South Australia and the University of Melbourne manage the screening of volunteers, using a two-step process: i) via an online survey using REDCap (Research Electronic Data Capture) [49] and, if eligible, ii) via in-depth telephone screening by the site-specific trial coordinators. Additional clearance to participate is sought from a general practitioner for anyone who does not pass exercise safety screening questions. The study interventions are undertaken at private, community physiotherapy clinics and provided by designated, trained trial physiotherapists.

### Data collection and management

Data are collected via: paper or online questionnaires completed by the participant (self-reported outcomes); paper and online data forms completed by the research personnel (objective assessment data); and accelerometry data from a wrist-worn monitor (assessment of physical activity levels). All online questionnaires/data forms are completed in REDCap. Paper questionnaires are stored in a locked cabinet and the data are manually entered into REDCap (self-reported outcomes) or double-entered into an excel spreadsheet (objective assessment data) by an outcome assessor. All digital data (questionnaires, objective assessment data forms, accelerometry data) are stored on a secure password-protected server. For all data collection, the ‘study knee’ is the painful knee, or in those with bilateral symptoms, the most painful eligible knee. If pain levels are similar bilaterally, the right knee is considered the study knee.

### Randomisation allocation concealment and blinding

Following completion of the baseline assessments (questionnaires, objective assessment, accelerometry), participants are randomised to either the EPIPHA-KNEE group or the Best Practice Education/Activity Control group. The computer-generated randomisation schedule was prepared by the study biostatistician using Research Randomizer [50], with randomly selected permuted blocks of varying size (4 and 6) and using a 1:1 ratio to the two treatment groups within four strata (sex and site: Adelaide, Melbourne). The EPIPHA-KNEE intervention and the Best Practice Control intervention are provided by different physiotherapists located at different clinic sites. Thus, once allocated to an intervention group, participants choose one of the candidate physiotherapy clinics that provide that intervention to attend (i.e., the clinic that is most convenient for them).

Allocation is concealed using central automated allocation, with security in place to ensure data cannot be accessed or influenced. The randomisation schedule is stored within a password-protected electronic system (REDCap). A hard copy of the raw randomisation schedule is also stored in a secure location at each site should the electronic system fail. Only the unblinded Research Leads (TRS in Adelaide, KB in Melbourne) have access to raw copies of the randomisation schedules. The trial coordinators at each site (Adelaide, Melbourne), randomise participants to intervention group. Notably, while the trial coordinators determine if a participant is eligible for inclusion in the trial, due to inaccessible online randomisation with use of randomly permuted block size and stratification, they are unaware when this decision is made to which group the participant will be allocated. Once randomised, the (now) unblinded trial coordinators at each site (Adelaide, Melbourne) coordinate treatment appointment scheduling with the appropriate physiotherapist as identified by the participant.

Many of the primary and secondary outcomes are self-report questionnaires, thus participants are also assessors for these outcomes. Participants are advised that they will be randomised to receive one of two active physiotherapy treatments involving education and exercise that aim to improve overall health (i.e., limited disclosure). The exact details of the interventions are not provided prior to randomisation. After randomisation, participants are provided only with details of the intervention they receive. Given that both groups receive active treatments and that the primary outcomes of the trial are not disclosed, we anticipate that this will be sufficient for blinding the participants to group assignment. At study completion, participants are asked to guess which group they were in (intervention group of interest or control group) in order to assess participant blinding.

For objective outcomes assessed in-person at baseline and at 12 weeks, research personnel whose roles are independent of treatment allocation and delivery (i.e., blinded to group allocation) act as the outcome assessors. Participants are explicitly instructed not to discuss their intervention or treating therapist with the outcome assessor. The same assessor evaluates the baseline and 12-week outcomes for each participant. To formally evaluate assessor blinding, the assessors are asked to guess which group the participants were in (intervention or control) after the participant’s 12-week objective assessment.

Treating therapists are unavoidably aware of the intervention they provide, but each therapist is assigned to deliver only one intervention only, and both groups are providing an active intervention. Our goal is to have therapists of both groups believe they are providing the intervention of interest; this will be formally assessed at completion of the trial. Regardless, therapists will not be involved in any outcome assessment. An independent, blinded statistician will perform data analyses.

### Intervention groups

**Fig. 2:**
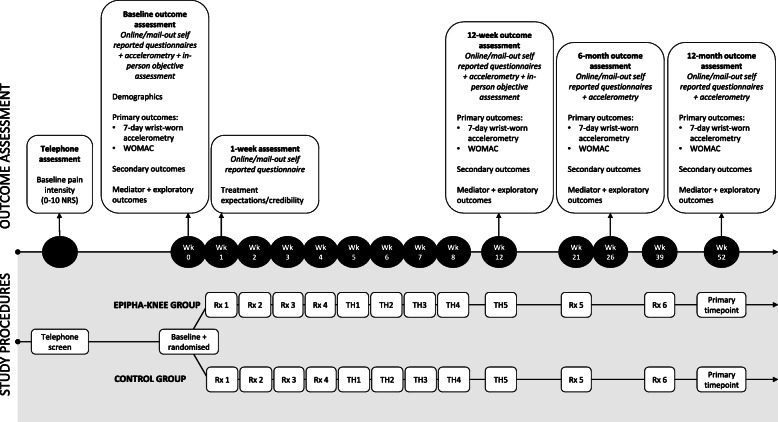
Study procedures and timing of outcome assessments. NRS = Numeric Rating Scale; Rx = in-person treatment session; TH = Telehealth (Telephone call or videoconferencing); Wk = Week; PSE = Pain Science Education; WOMAC = Western Ontario and McMaster Universities Osteoarthritis Index

Both intervention groups receive consistent, standardised general OA/activity education, graded walking and strengthening components, but have a contrasting OA pain education component. The EPIPHA-KNEE group receives contemporary PSE underpinned by principles of self-regulated learning and conceptual change theory [29, 51–53] and modified to integrate contemporary biological science of OA [14, 16, 17, 43, 44]. The Best Practice Control group receives best practice OA education, consistent with OA management clinical practice guidelines [31]. Table 2 highlights the different aspects of the educational component and Fig. 2 illustrates the timing of the intervention components and assessment points. Participants in both groups keep a weekly paper-based diary of education, walking, strengthening, and any homework activities to promote improved adherence [54**],** and in the EPIPHA-KNEE group, the diary is also used to promote self-regulated learning via guided and independent reflection upon what was achieved [51].

**Table 2:** Education features of the intervention groups

	Enhanced PSE – EPIPHA-KNEE	Standard education – Control
**Overall objective(s):**	To shift participants’ conceptualisation of pain from that of a marker of tissue damage to that of a marker of the perceived need to protect the body. To educate that pain is a protective feature of our system, not a ‘damage-meter’; thus, pain can be modulated by other things besides tissue damage and danger messages (i.e. nociception). To understand the three key ingredients to recovery in OA: i) increasing knowledge; ii) increasing activity; iii) reducing inflammation.	To increase participants’ knowledge about OA and the importance of physical activity in reducing osteoarthritic pain and improving general health.
**Pain education topics:**	Basic nervous system anatomy/function; distinction between nociception and pain; protective function of pain; peripheral/central sensitisation; up-regulation of brain mechanisms that serve protection; the state of ‘hyper-protection’ offered by normal biological adaptations; the concept of an internal ‘Protectometer’ (modulated by multifaceted danger and safety cues); the concept of bioplasticity (all tissues and systems adapt and are changeable, including cartilage, bone, muscles, the pain system, the immune system, etc.); the importance and contribution of body wide inflammation to knee symptoms.	Basic OA and pain information as per the Arthritis Australia handbook.
**Activity education:**	That physical activity does not increase joint damage but does have wide-ranging health benefits and OA-specific benefits. That physical activity is key to bioplasticity – i.e., inducing change in our system – and that activity decreases the over-protectiveness of the pain system (clarifying that over-protectiveness is a change that often occurs with persistent knee pain). That the aim of graded activity is to slowly increase loading of the joint and body systems; introducing the concept of the sweet zone (the optimal amount of activity that is not too much nor too little, but sufficient to promote bioplasticity).	That physical activity has wide-ranging health benefits as well as OA-specific benefits and that even people with severe OA benefit from activity.
**X-Ray interpretation**^a^**(if applicable):**	The aim is to provide education about *why* scans are no longer recommended for diagnosis or to guide treatment of OA (i.e., scans do not provide sufficient or valuable information about current or future pain/function; clinical symptoms provide more reliable information). Therapists will ask participants if they have had a scan of their knee. If they have had a scan, the aim is to ‘de-threaten’ radiological findings through asking participants about what they have been told about their scans, and what information they thought scans provided them about their knee and their activity (exploring how they feel – e.g., fearful, anxious). It will also include discussing the report, focusing on positive features (e.g., normal age-related changes). If they have not had a scan, the aim is to reassure the participant that they do not need one. In both cases, education about the poor correlation between x-ray findings and pain will be provided.	The aim is to explain that scans are no longer recommended for diagnosis or to guide treatment of OA, consistent with contemporary clinical practice guidelines. Therapists will ask participants if they have had a scan of their knee. If they have had a scan, the aim is to discuss radiological findings, focusing on the interpretation section as would occur in regular practice.
**Walking program**	The aim is to use the concept of an internal ‘Protectometer’ in pain to purposefully vary and embed context into the graded walking program that is individualised to the participant and their unique goals.	The aim is to educate participants about the need for graded increases in walking when performing an individualised program aimed towards their unique goals.
**Strengthening program**	The aim is to have participants reflect on the strengthening program and its effect on knee strength/stability and on confidence in moving. The aim is also to apply the idea of a ‘Protectometer’ to strengthening exercises, by varying and embedding context into their performance.	The aim is to educate participants about the importance of continued and regular strengthening exercise of the main lower limb muscle groups to assist in maintaining knee function.

PSE = Pain Science Education. ^a^All standard educational features will also occur in the EPIPHA-KNEE group, with the exception of standard x-ray interpretation

#### EPHIPHA-KNEE group (enhanced PSE integrated with general education, graded walking program and strengthening program)

Participants attend four, 60–90 min, in-person individual sessions with a physiotherapist at weekly intervals (over 4 weeks), during which they participate in enhanced PSE (“Explaining Pain”) as well as receiving a graded walking program and strengthening exercises, which both integrate PSE concepts. All in-person sessions take place at the private physiotherapy clinic where the trial physiotherapist is employed. The in-person sessions are followed by 4 weeks of at-home activities, involving workbook activities and walking/strengthening progression, with weekly telephone calls or videoconferencing sessions (via Zoom) by the physiotherapist (20 mins each). A follow-up telehealth session at 3 months (20 mins) and follow-up in-person sessions with the physiotherapist (45–60 min) occur at 5 and 9 months. A detailed program plan, including learning objectives, tasks and assessments has been created for this intervention (currently embargoed, but the base curriculum and trial-specific therapist intervention case notes and participant diaries which include at-home activities have been uploaded and time-locked on Open Science Framework [55], see: osf.io/cs2rx). Participants receive a custom-made EPIPHA-KNEE handbook (Explain Pain Knee Osteoarthritis Handbook) [56] to take home, which serves as a reference point and activity manual to consolidate and further promote learnings. All educational concepts are covered with all participants, but therapists personalise the educational content to the individual (e.g., time spent on concept; linking discussion of content to the participant’s circumstances and using the participant’s own wording when possible). The walking and strengthening programs are personalised to the participant, but are guided by a standardised protocol of graded increases. Choice of telephone versus videoconference for at-home telehealth sessions is at the participants’ preference.

#### Best practice education/activity control group (standard OA education, graded walking program and strengthening program)

Participants attend four, 45–60 min, in-person sessions with a qualified physiotherapist at weekly intervals (over 4 weeks), during which they receive general information about OA and activity as well as receiving a graded walking program and strengthening exercises. All in-person sessions take place at the private physiotherapy clinic where the trial physiotherapist is employed. The in-person sessions are followed by 4 weeks of at-home activities (workbook and walking/strengthening progression) with weekly telephone calls or videoconferencing sessions (via Zoom) by the physiotherapist (20 mins each). A follow-up telehealth session (3 months) and follow-up in-person sessions with the physiotherapist (30–45 min) occur at 5 and 9 months. Participants receive the Arthritis Australia ‘Taking control of your osteoarthritis’ booklet [57] to take home, which discusses educational concepts for this intervention. Full therapist case notes are embargoed but have been uploaded and time-locked on Open Science Framework (see: osf.io/cs2rx). The walking and strengthening programs are personalised to the participant but are guided by a standardised protocol of graded increases. Choice of telephone versus videoconference for at-home telehealth sessions is at the participants’ preference.

#### Graded walking program

The walking program is based on that previously used in our feasibility study [42]. The current trial’s *minimum* activity goal for all participants at 12 months is to walk at least 30 min, 5 days per week at a moderate intensity (defined as being moderately out of breath while walking), consistent with physical activity guideline recommendations [58]. There is no maximum activity goal; this is individualised to the participant, and if appropriate, can include activities such as hiking or jogging, for longer than 30 min and for up to 7 days per week at moderate or vigorous intensity. The planned walks are guided by principles of pacing, including graded increases over time, goal-setting, as well as pre-planned (times/dates) walks, with the aim to complete the walking goal regardless of how they feel (unpair activity and pain associations) [59]. Further, graded increases are limited to one factor per week: walking duration; number of walks; walking intensity; or other features (hills, differing terrain). Thus, if walking duration is increased, the number of walks and their intensity (and other features) are held constant that week. This program has been visually depicted in a past publication [42] and is summarised here in Table 3. All walking program features, individualised participant goals, and walking progression principles are identical between groups. Table 2 highlights how the PSE component is embedded within the walking program for the EPIPHA-KNEE group.

**Table 3:** Summary of walking program

Time point	Objectives	Details/progression strategies
***OVERALL MINIMUM WALKING GOAL***		***30 min duration, 5–7 days/week at a moderate intensity (moderately out of breath, moderately difficult to talk)***
**WEEK 1**	Find baseline walking tolerance (3–4 walks to tolerance)	Each participant’s unique walking tolerance is determined by having the participant take 3–4 walks during the following week (number of walks individualised to the participant by therapist), and recording how far (time/distance) they can walk before they experience a significant increase in knee symptoms (operationalised as a significant increase in their knee pain [≥2 points on an 11-point NRS) or knee swelling [visually noticeable) or pain/swelling that lasts [~ 2 h] after they finish walking. ***In Week 1–4 (weekly in-person sessions), the therapist guides the walking program and assists the participant in planning the dates and times that the walks will be undertaken.***
**WEEK 2**	“Start” walking level (80% of baseline tolerance, 4–5 walks) Goal setting	The therapist takes an average of the walking time/distance over the 3–4 baseline walks, with 80% of this time/distance used as the “start” walking level for the following week, but with an additional day of walking (e.g., if 3 walks performed in Week 1, Week 2 involves 4 walks at 80% of Week 1’s time/distance). Facilitated, individualised goal-setting: the therapist assists the participant in creating short- and long-term activity-related goals (using Specific, Measurable, Achievable, Relevant, Time bound [SMART] format). These goals are used to help individualise the subsequent walking program.
**WEEK 3**	Progress 10% (90% of baseline walking tolerance, 4–5 walks)	The participant progresses to 90% duration of their baseline walking tolerance, keeping the total number of walks constant (i.e., 4–5 walks).
**WEEK 4**	Progress 10% (100% of baseline walking tolerance, 4–5 walks) Goal review	The participant progresses to 100% duration of their baseline walking tolerance, keeping the total number of walks constant (i.e., 4–5 walks). Short- and long-term goals are reviewed.
**WEEK 5–8**	Progress 10% or increase number of walking days Goal review (Week 8)	Weekly progressions either by increasing the walking duration by 10%/week or by adding in additional walking days, but not both. The choice to add duration or walking days is individualised to the participant and guided by the minimum activity goal of the trial. ***In Week 5–8 (weekly telehealth sessions), the physiotherapist assists the participant in updating the walking program, with the aim of having participants able to independently progress their own program*.** At Week 8 (telehealth session), the therapist assists the participant in planning activity for the subsequent weeks until the Week 12 session, and the short- and long-term goals are reviewed.
**WEEK 9–12**	Progress 10% or number of days or additional progression strategies (see below) Goal review (Week 12)	After the minimum activity goal of the trial is met in terms of duration and walking days, intensity is increased by increasing walking speed (if applicable). Individualised progression can also occur in terms of types of walks, progression to jogging. ***If a flare-up is experienced (at any time), the therapist assists the participant in appropriately revising the walking program.*** At Week 12 (telehealth session), the therapist assists the participant in planning activity for the subsequent weeks (in-person session at Week 21) and the short- and long-term goals are reviewed.
**Week 13–52**	Progression strategies or maintenance Goal review (Week 21, 39)	Individualised progression strategies are undertaken. If participant has met the minimum activity goal and does not want to further increase activity, maintenance planning is undertaken. At Week 21 and 39 (5 and 9 month in-person follow-up physiotherapy sessions), the therapist assists the participant in planning activity for the subsequent weeks and the short- and long-term goals are reviewed.
**ADDITIONAL PROGRESSION STRATEGIES**	To individualise progression to the individual and their personal activity goals.	Walking intensity: Walking speed is increased, using breath and ease of talking as a guide for intensity (low intensity: minimally out of breath and easy to talk; moderate intensity: moderately out of breath, moderately difficult to talk; vigorous intensity: out of breath, difficult or unable to talk). Initiation of new walking activities (e.g., hiking up hills, walking on different terrain): Baseline tolerance is first evaluated using 3–4 walks over a week, followed by initiation of 4–5 walks the following week at 80% of tolerance. Alternatively, different types of walks (hills, differing terrain) may also be integrated within normal walks (3 normal walks, two hill walks/week). Jogging: Baseline tolerance is evaluated by interspersing 1 min jogging with 1 min walking intervals. Relative jogging time is progressively increased over weeks (e.g., week 2: 2 min jogging, 1 min walking; week 3: 3 min jogging, 1 min walking), keeping the total duration constant. Once the participant can jog for the entire baseline duration, then jogging time/distance/intensity can be increased.

### Graded strengthening program

The strengthening program received by both intervention groups involves general lower limb strengthening exercises that are individualised to the participant and progressed in dosage and in difficulty over time. The exercises chosen are based on those used in previous trials by the research team which were found to be effective [60]. Table 4 provides the strengthening exercises and a proposed progression over 12 weeks but is used as a guide only. Physiotherapists use information from their subjective and objective assessment to individualise the strengthening exercise program to their participant (e.g., can start with more difficult exercises if needed). Participants are provided with a handout that visually depicts each exercise and provides key instructions about performance (see Supplementary File 1). Using the handout as a reference, physiotherapists demonstrate each exercise to the participant during the in-person sessions (during Session 2 and Session 4) and then observe the participant performing the exercise, providing feedback as needed. When possible (and with consent), physiotherapists also video the participant performing each of the exercises correctly on the participant’s phone so that the participant can refer to the video in the future (e.g., during at-home sessions) if needed. After week 13, continuation of the strengthening program is left open to each participant; however, if continued, no further exercise progression occurs beyond which is stipulated in Table 4. Table 2 highlights how the PSE component is embedded within the strengthening program for the EPIPHA-KNEE group.

**Table 4:** Strengthening exercise programme – ideal prescription Weeks 1–12

Week	Strengthening exercise prescription (completed the week after the session)
***In-person Treatment Sessions***	
**1**	**No exercises.** Establishing baseline walking tolerance (current capacity)
**2**	**Wall squats:** 1 set of 10 repetitions, 2 times this week
	**Sliding (forwards/backwards):** 1 set of 10 repetitions, 2 times this week
**3**	**Wall squats:** 2 sets of 10 repetitions with 30–60 s break between sets, 2 times this week
	**Sliding (forwards/backwards):** 2 sets of 10 repetitions with 30–60 s break between sets, 2 times this week
**4**	**Wall squats:** 3 sets of 10 repetitions with 30–60 s break between sets, 2 times this week
	**Sit-to-stand:** 1 set of 10 repetitions, 2 times this week
	**Stepping (forwards/backwards):** 1 set of 10 repetitions, 2 times this week
	*If sliding not going well, do not replace with stepping.
	***IMPORTANT: Demonstrate and video all remaining exercises.
***Phone Calls/Telehealth Sessions***	
**5**	**Sit-to-stand:** 2 sets of 10 repetitions with 30–60 s break between sets, 2 times this week
	**Stepping (forwards/backwards**): 2 sets of 10 repetitions with 30–60 s break between sets, 2 times this week
**6**	**Sit-to-stand:** 3 sets of 10 repetitions with 30–60 s break between sets, 2 times this week
	**Stepping (forwards/backwards):** 3 sets of 10 repetitions with 30–60 s break between sets, 2 times this week
	*If doing well, progress stepping to step-ups and step-downs
**7**	**Sit-to-stand:** 3 sets of 10 repetitions with 30–60 s break between sets, 2 times this week
	**Step-ups (low step):** 1 set of 10 repetitions, 2 times this week; *use low step, monitor carefully*
	**Step-downs (low step):** 1 set of 10 repetitions, 2 times this week; *use low step, monitor carefully*
**8**	**Step-ups (low step):** 2 sets of 10 repetitions with 30–60 s break between sets, 2 times this week
	**Step-downs (low step):** 2 sets of 10 repetitions with 30–60 s break between sets, 2 times this week
	*If step-ups not well tolerated continue with stepping; if step-downs not well tolerated continue with Sit-to-Stand.
	*If applicable, discuss future use of a higher step (and source this) with participants.
***Participant doing at-home exercises; no scheduled contact with participants***	
**9**	**Step-ups (low step):** 3 sets of 10 repetitions with 30–60 s break between sets, 2 times this week
	**Step-downs (low step):** 3 sets of 10 repetitions with 30–60 s break between sets, 2 times this week
	*If step-ups not well tolerated continue with stepping; if step-downs not well tolerated continue with Sit-to-Stand.
**10**	**Step-ups advanced (increased step height):** 3 sets of 10 repetitions total (1 set at increased step height) with 30–60 s break between sets, 2 times this week
	**Step-downs advanced (increased step height):** 3 sets of 10 repetitions total (1 set at increased step height) with 30–60 s break between sets, 2 times this week
	*If step-ups/step-downs advanced are not well tolerated continue with normal step-ups.
**11**	**Step-ups advanced:** 3 sets of 10 repetitions total (2 sets at increased step height) with 30–60 s break between sets, 2 times this week
	**Step-downs advanced:** 3 sets of 10 repetitions total (2 sets at increased step height) with 30–60 s break between sets, 2 times this week
	*If step-ups/step-downs advanced are not well tolerated continue with normal step-ups.
***Phone Call/Telehealth Session***	
**12**	**Step-ups advanced:** 3 sets of 10 repetitions (all at increased step height) with 30–60 s break between sets, 2 times this week
	**Step-downs advanced:** 3 sets of 10 repetitions (all at increased step height) with 30–60 s break between sets, 2 times this week
	*If step-ups/step-downs advanced are not well tolerated continue with normal step-ups.
**13 onwards**	Strengthening exercises optional to continue

### COVID-19 considerations

Should COVID-19 restrictions return that prevent the provision of physiotherapy treatment in-person, the interventions of both groups will be updated such that all in-person sessions are instead provided via telehealth (either telephone call or videoconferencing via Zoom). Further, if COVID-19 restrictions return that limit outdoor physical activity, both groups will be provided with ideas for maintaining their activity levels within their available spaces. All COVID-19 restrictions for Adelaide and Melbourne, and the dates of implementation, will be documented throughout the trial period. Lastly, if COVID-19 restrictions prevent in-person objective assessments (baseline, 12 weeks) that take place at the University of South Australia and the University of Melbourne, study participation will proceed without these assessments. These steps aimed at trial flexibility are consistent with recommendations made to preserve trial integrity during the COVID-19 pandemic [61].

### Physiotherapist training

A minimum of twelve physiotherapists per group (aim of *n* = 6/group per site) are employed to provide the intervention. The trial’s EPIPHA-KNEE intervention is provided by physiotherapists with a minimum of 5 years clinical experience who have undertaken high-level pain education training (Explain Pain course; NOIgroup). The trial’s control intervention is also provided by physiotherapists with a minimum of 5 years clinical experience, but who have not undertaken high-level pain education training.

Prior to trial commencement, physiotherapists in the EPIPHA-KNEE group complete intensive training in PSE specific to OA (i.e., the EPIPHA-KNEE intervention), involving pre-reading of key papers (4 h) and attendance at two full days (16 h) of in-person group training. The training days are delivered by leading experts in pain science, health education, and osteoarthritis. The training includes orientation to the content of the educational component of the trial intervention (i.e. educational objectives for participants), and advanced training in skills to promote conceptual change and self-regulated learning.

Physiotherapists in both intervention groups also complete online training modules (5.5 h EPIPHA-KNEE group, 4 h Control group) for: i) trial procedures (including key trial contacts, mandatory reporting to research team, ethical and confidentiality matters); ii) the relevant educational intervention (including specific objectives for each treatment session and instructions on how to individualise education to the participant); iii) the graded walking program (the guiding principles, ideal prescription, how to individualise to the participant, and how to vary if flare-ups occur); iv) the strengthening exercise program (as per walking program); v) intervention delivery (how to integrate the three components of Education, Walking program, and Strengthening program within each session, how to use the Case Notes); vi) telehealth sessions (tips to ensure telehealth runs smoothly). Lastly, formal competency checks of the physiotherapists in both groups are undertaken by the research team via: i) review of physiotherapist’s verbal/written answers to questions about the trial protocols and the interventions; ii) auditing of (and feedback on) audio-recorded intervention sessions for a pilot participant treated by each physiotherapist.

### Treatment fidelity

Following trial commencement, the trial physiotherapists can discuss any issues that arise with delivering the intervention with the research team. Trial physiotherapists can contact the research team through the trial by email or telephone. Additionally, regular meetings via Zoom are held with the trial research team and each intervention group of physiotherapists (separately). These regular meetings also allow the opportunity for therapists to provide feedback or discuss any issues arising. A random sample of treatment sessions (minimum of one for each therapist) will be audio recorded to allow an audit of intervention fidelity for each group. Each physiotherapist and physiotherapy clinic will provide only one of the two study interventions. Therapists’ case notes for each session, recorded via standardised templates, will also be used to evaluate intervention fidelity (e.g., the exercises and walking prescribed).

### Dealing with adverse events

Given the provision of walking and strengthening exercises, we expect transient increases in lower limb pain or swelling (including, but not limited to, the trial knee) due to increased structured physical activity. Trial therapists and participants are advised to report any adverse events to the study coordinator as soon as possible via documentation in treatment notes uploaded online in REDCap (therapists) as well as via email or telephone call to the study coordinator (therapists and participants). If needed, trial participation is continued in a revised manner (including the potential for additional treatment sessions for the problem), or treatment is discontinued and further medical intervention is arranged. Adverse events are formally collected via participant self-report following weekly intervention completion (12 weeks) and at the 6 and 12 month follow-up questionnaires.

### Outcomes

Table 5 provides the full list of outcome measures and time points of assessment. Our primary and secondary outcome measures are measured at baseline, 12 weeks, 6 and 12 months.

**Table 5:** Summary of measures to be taken

Domain	Data collection instrument	Time points				
		Baseline	After 1st treatment session	12 weeks	6 M	12 M
**Descriptive data**						
Age, gender, weight, height, body mass index		X				
Duration of knee OA symptoms		X				
Educational level, postcode		X				
Treatment and medication use in past month		X		X	X	X
Problems in other joints		X				
Co-morbidities	Functional Co-morbidity Index	X				
**Primary outcome measures**						
Physical activity level	Average daily step count via wrist-worn accelerometry	X		X	X	X
OA knee symptoms in past 48 h	WOMAC Total score (includes pain, physical function, stiffness subscales)	X		X	X	X
**Secondary outcome measures**						
Average knee pain past week	11-point NRS	X		X	X	X
Average knee pain during walking past week	11-point NRS	X		X	X	X
Physical activity level	Daily minutes of sedentary, light, moderate, vigorous activity via wrist-worn accelerometry	X		X	X	X
Knee pain past 48 h	WOMAC pain subscale	X		X	X	X
Knee function past 48 h	WOMAC function subscale	X		X	X	X
Depression past week	4-item PROMIS depression subscale	X		X	X	X
Anxiety past week	4-item PROMIS anxiety subscale	X		X	X	X
Stress past month	4-item PSS	X		X	X	X
Fear of movement	Brief FOM questionnaire	X		X	X	X
Pain catastrophising	PCS	X		X	X	X
Health related quality of life	EQ-5D-5L	X		X	X	X
Knee perception	FREKAQ	X		X	X	X
Global rating of change	7 point ordinal scale			X	X	X
**Mediators**						
OA/activity Conceptualisation	Osteoarthritis and Physical Activity Conceptualisation Scale	X		X	X	X
Pain self-efficacy	PSEQ	X		X	X	X
Exploratory: Implicit movement bias	Implicit association task	X		X		
Exploratory: Implicit environmental bias	Distance estimation task Hill steepness estimation task	X		X		
Exploratory: Pain sensitivity to activity	Pain pre−/post- 6 min walk task	X		X		
**Exploratory objective factors and questionnaires (potential modulators/moderators)**						
Implicit motor imagery	Left/right judgement task	X		X		
Tactile acuity at the knee	Two point discrimination task	X		X		
OA pain conceptualisation	OA pain drawing task	X				X
Self-regulated learning ability	Items from the Motivated Strategies for Learning and Regulation of Learning questionnaires	X		X	X	X
**Resource use**						
Health care use^a^	Resource use associated with the delivery of the intervention. Self-reported use of health services and co-interventions; MBS; and hospital admissions and emergency department presentations from administrative datasets	X		X	X	X
Medication use^a^	Self-reported medication use and PBS data from administrative datasets	X		X	X	X
**Additional outcomes**						
Expectation of treatment outcome	5 point ordinal scale		X			
Treatment credibility	Modified Treatment Credibility/Expectancy Questionnaire		X			
Intention to exercise	7 point ordinal scales (commitment, motivation, determination to exercise)		X	X	X	X
COVID-19 impact on physical activity	7-point ordinal scale	X		X	X	X
Adherence	Self-rated adherence to walking and strengthening program, 11-point NRS and number of days in past week exercise performed			X	X	X
	Number of in-person sessions attended			X	X	X
	Number of telehealth sessions attended			X		
	Review of patient diary					X
Harms	Adverse events			X	X	X
Blinding	Participant blinding assessed via allocation guesses (and reasons); then forced choice					X
	Outcome assessor blinding (same method)			X		
	Therapist blinding (same method)					X^b^
Therapist related factors	Beliefs about exercise; self-rated via Therapist Beliefs about Exercise Questionnaire	X				X^c^
	Beliefs about pain; self-rated via Pain Attitudes and Beliefs Scale for Physiotherapists	X				X^c^
	Pain knowledge; assessed via rNPQ	X				X^c^
	Perceived intervention credibility; assessed via modified intervention credibility and expectancy questionnaire	X		X^d^		X^d^
	Perceived connection with participant; self-rated via 101-point NRS Connectedness Scale			X		

^a^Data on MBS, PBS and emergency department presentations and hospital admissions will be sourced from government-held administrative datasets for the entire 12 month follow-up period at its conclusion. ^b^Measured at end of trial (recruitment complete); aim is to determine if the ‘Best practice control group’ therapists thought they were the intervention group of interest. ^c^Measured at baseline (pre-training), post-training, and end of trial (recruitment complete). ^d^Measured after pre-trial training (baseline), after the first 12 weeks of treatment (pilot participant), mid-way through the trial (i.e., half-way through recruitment), and at the end of the trial (recruitment complete)

### Primary outcomes

The primary outcomes are: i) average daily step count assessed over 7 consecutive days via wrist-worn accelerometry (Actigraph GT9X Link, Pensacola, FL); and ii) overall knee symptoms measured via the Western Ontario and McMaster Universities (WOMAC) Osteoarthritis Index [62]. The primary timepoint for both outcomes is 12 months.

For daily step count, participants wear the accelerometer on their non-dominant wrist at all times (removal only for shower or during water-based activities). Data are collected from the device in 60 s epochs. A paper-based activity log is provided to participants to capture times they go to bed at night and get up from bed in the morning, as well as times the accelerometer was removed and then placed back on (particularly if taken off when sleeping). Valid wear time is operationalised as having ≥10 h of waking wear time for a minimum of 4 days (3 weekdays and 1 weekend day) [63, 64]. Average daily step count is calculated as the weighted average (5:2) of weekday and weekend step counts. We chose daily step count as a primary outcome based on its: i) health benefits in knee OA (increased physical activity via walking improves both pain and function [65], and even a moderate increase in physical activity [1000 steps/day] reduces risk of functional decline [66]); and ii) established reliability/validity in knee OA [67].

The WOMAC is a disease-specific self-report questionnaire that includes pain (5 items), stiffness (2 items), and physical function (17 items) subscales. Items are scored from 0 to 4, giving a range of possible scores from 0 (no symptoms or dysfunction) to 96 (maximal symptoms and dysfunction). It has demonstrated validity, reliability, and responsiveness [68]. The use of the total score for primary outcomes is supported by the scale creators.

### Secondary outcomes

The secondary outcome measures are:

i) Average knee pain over the past week rated using an 11-point Numerical Rating Scale, anchored with “0, no pain at all” to “10, worst pain possible” [47];
ii) Average knee pain while walking over the past week using an 11-point Numerical Rating Scale, anchored with “0, no pain at all” to “10, worst pain possible” [47];
iii) Knee pain over the past 48 h, via the WOMAC 5-item pain subscale score, with items scored from 0 to 4, giving a range of possible scores from 0 (no pain) to 20 (maximal pain) [62];
iv) Knee function over the past 48 h via the WOMAC 17-item function subscale scores, with items scored from 0 to 4, giving a range of possible scores from 0 (no dysfunction) to 68 (maximal dysfunction) [62];
v) Depression, assessed using the 4-item PROMIS depression subscale, with items scored on a 5-point Likert scale from “Never” (1) to “Always” (5), giving a range of scores from 4 to 20 (rescored using a T-score metric with a score of 50 representing the mean and 10 the standard deviation of a reference population), with higher scores reflecting greater depression [69];
vi) Anxiety, assessed using the 4-item PROMIS anxiety subscale, with items scored on a 5-point Likert scale from “Never” (1) to “Always” (5), giving a range of scores from 4 to 20 (rescored using a T-score metric with a score of 50 representing the mean and 10 the standard deviation of a reference population), with higher scores reflecting greater anxiety [69];
vii) Stress over the past month, assessed using the 4-item Perceived Stress Scale, scored on a 5-point Likert scale from “Never” (0) to “Very often” (4), giving a range of possible scores of 0 (no perceived stress) to 16 (maximal perceived stress) [70, 71];
viii) Fear of movement, assessed using the 6-item Brief Fear of Movement for OA scale, scored on a 4-point Likert Scale from “Strongly disagree” (0) to “Strongly agree” [3], giving a range of possible scores of 0 (no fear of movement) to 18 (maximal fear of movement) [19];
ix) Pain catastrophising, using the brief 4-item Pain Catastrophising Scale (PCS), scored on a 5-point Likert scale from “Not at all” (0) to “All the time” (4), giving a range of possible scores of 0 (no pain catastrophising) to 16 (maximal pain catastrophising) [72];
x) Health related quality of life (HrQOL) using the EQ-5D five level instrument (EQ-5D-5L scale) [73]. The EQ-5D-5L measures HrQOL across five dimensions (mobility, self-care, usual activities, pain/discomfort and anxiety/depression) each with five possible response options ranging from no problems (1) to unable or extreme problems (5). It also includes a 100-point global health rating using a visual analogue scale (EQ-VAS) ranging from 0 (worst imaginable health) to 100 (best imaginable health).
xi) Knee perception using the Fremantle Knee Awareness Questionnaire (FREKAQ), involving 9-items, scored on a 5-point Likert scale from “Never” (0) to “Always” (4) with giving a range of possible scores of 0 (no perceptual dysfunction) to 36 (maximal perceptual dysfunction) [74];
xii) Global rating of change, scored on a 7-point Likert scale for “Overall change in your study knee since you began the study”, “Overall change in physical activity”, “Overall change in pain”, and “Overall change in physical function” from “Much worse” to “Much better”. Responses will be dichotomised to “Improved” (“Moderately better” and “Much Better”) and “Not improved” (all other choices) [75];
xiii) Average daily minutes of sedentary, light, moderate, and vigorous activity and volume (intensity x time) from the wrist-worn accelerometry using two published methods for cut-point-based analysis: Troiano cut-points [63, 76], and those from Smuck et al. (2017) [77].

### Demographic and descriptive data

At baseline, the following data are collected: i) age; ii) sex; iii) height and weight; iv) postal code; v) educational level; vi) duration of symptoms, vii) presence of co-morbidities (via the Functional Co-morbidity Index [78]), viii) pain in others parts of the body, ix) medication use over the past month; and x) interventions received during the past month.

### Mediators

#### Primary mediators

i) Conceptualisation of OA and physical activity, assessed using the Knee OA and Activity Conceptualisation Scale (OACS), which includes 33 items scored on a 5-point Likert scale from “Strongly agree” (1) to “Strongly disagree” (5), with scores ranging from 33 (low conceptualisation) to 165 (high conceptualisation);
ii) Pain self-efficacy assessed using the Pain Self-efficacy Questionnaire (PSEQ) [79], which includes 10 items scored on a 7-point Likert scale from “Not at all confident” (0) to “Completely confident” (6), with scores ranging from 0 (no confidence to perform activity despite pain) to 60 (maximal confidence to perform activity despite pain).

### Exploratory mediators (if sufficient data are available)

i) Implicit movement bias: An implicit association test based upon established methodology [80] will be used to evaluate the presence of positive or negative movement bias. Using 2 target categories (image of knee movement – ‘active’ vs image of knee resting – ‘rest’) and 2 attribute categories (‘safe’ and ‘danger’), participants sort images into categories: responses are faster/with fewer errors if the target and the attribute category are associated for that person, and are slower/with more errors if they are not [80], allowing determination of negative movement bias (faster movement–‘danger’ and rest–‘safe’ pairings) and positive movement bias (faster movement–‘safe’ and rest–‘danger’ pairings).
ii) Implicit environmental bias: Perceptual shifts in relation to the environment will be assessed via estimation of the distance to a target for a walking task [81] and via estimation of hill steepness [82], the latter using virtual reality (equivalent responses to real-life) [45]. Errors between estimated and actual distance/steepness are used.
iii) Pain sensitivity to activity, assessed via calculating the change in knee pain intensity ratings (11-point NRS) from pre- to post-standardised 6 min walk test [83].

### Additional measures

#### Exploratory objective factors and questionnaires (potential modulators/moderators)

These include: i) implicit motor imagery performance, assessed via left/right judgement tasks (accuracy and response time) for images of feet and of hands (control) [84] using the Recognise App (NOIgroup Pty Ltd) on an iPad; ii) tactile acuity at the knee assessed using callipers to determine the two-point discrimination threshold at the medial knee [85]; iii) abstract OA pain conceptualisation as assessed using an OA pain drawing task whereby participants are asked to draw their understanding of OA and why it hurts; iv) Self-regulated learning ability, assessed using 7 items from the Motivated Strategies for Learning Questionnaire [86], with items rated on a 7-point Likert scale from “Not true at all for me” to “Very true for me”.

#### Treatment expectations/credibility

Expectation of treatment outcomes are assessed using a 5-point ordinal scale in response to the question “What effect do you think this treatment will have on your knee problem”, with choices from “no effect at all” to “complete recovery”. A modified Credibility/Expectancy Questionnaire (CEQ) [87] is also used to assess treatment credibility via 4 questions: “How logical does this treatment offered to you seem”; “How successful do you think this treatment will be in reducing your knee OA symptoms”; “How successful do you think this treatment will be in increasing how much you move”; “How confident would you be in recommending this treatment to a friend who experiences similar problems”. These are rated on a 9-point Likert scale from “not at all logical/useful/confident” to “very logical/useful/confident”.

#### Intention to exercise

Intention to exercise is assessed via 3 questions: “I am committed to engage in physical activity over the next 2 weeks”; “I am motivated to engage in physical activity over the next 2 weeks” and “I am determined to engage in physical activity over the next 2 weeks” [88]. A 7-point Likert scale from “very strongly disagree” to “very strongly agree” is used.

#### Measurement and valuation of resource use

Total costs associated with delivering the intervention, including resources used in a) the development and provision of face-to-face, evidence-based education sessions and b) the development and provision of home activities and weekly development goals, workbook and telephone contacts, will be measured and valued using appropriate unit costs. Health service utilisation data for the participants, including in-patient hospital stays and emergency department presentations will be accessed from relevant state-based health department administrative records. Individual-level Medicare Benefits Schedule (MBS) and Pharmaceutical Benefits Scheme (PBS) data (including specialist consultations, visits to general practitioners, and access to prescription pharmaceuticals) will be collected from administrative databases held by Services Australia following informed consent from trial participants. Any additional services accessed outside of these programs will be collected via participant self-report. Following relevant guidelines, costs for relevant healthcare resources will be sourced from publicly available published data (including the National Health Cost Data Collection, MBS and the PBS) [89–92].

#### Adverse events

Participants are asked to provide details on the nature of the event, how long it lasted, and what action they took. For the purpose of this trial we define adverse events as “any untoward medical occurrence in a patient or clinical investigation participant administered a treatment and which does not necessarily have a causal relationship with this treatment. An adverse event can therefore be any unfavourable and unintended sign (including an abnormal laboratory finding), symptom, or disease temporally associated with the use of a treatment, whether or not related to the treatment”. The trial physiotherapists (or study coordinators, if contacted by participants directly) record details of the adverse event, including whether it was related or unrelated to the study intervention.

#### Adherence

Adherence is assessed in each group via a review of attendance records for in-person physiotherapy appointments and telephone/videoconferencing physiotherapy sessions. Adherence to the prescribed walking programs is self-reported by the participant via 11-point Likert scale (0 means “strongly disagree” and 10 means “strongly agree”) in response to three statements: “I have been doing the number of walks each week as recommended”; “Within each walk, I have been walking the distance/time/steps as recommended”; and, “Within each walk, I have been walking at the recommended intensity”. Participants are also asked to indicate on how many days in the past week they walked for exercise (options of 0 to 7 days). Adherence to the strengthening program is also self-reported using similar assessment (“I have been doing the strengthening exercises each week as recommended”; “For each exercise, I have been doing the sets/repetitions as recommended”; and, “In the past week, on how many days did you perform the strengthening exercises?”). When available, adherence will also be informed via review of participant workbook, walking program, and strengthening program diaries that they keep over the 12 months.

#### COVID-19 impact on physical activity

Self-reported impact of COVID-19 on physical activity is assessed via the question, “During the past 4 weeks, has COVID-19 influenced your usual physical activity levels/exercise routines?” with dichotomous response options of “Yes” or “No”. If participants answer yes, they self-report the extent to which COVID-19 has influenced usual physical activity levels/exercise routines on a 6-point Likert scale ranging from “Large reduction” to “Large increase”. Additionally, trial coordinators in Adelaide and Melbourne keep weekly records of the state recommendations and/or restrictions in relation to travel, physical activity, use of public transport, ability to leave the home, etc.

#### Blinding

Following an explanation to participants that clinical trials typically have an intervention of interest (active intervention) that is tested to see if it is better than the other intervention (control intervention), they are asked to identify which treatment they believe they received (choices: “Active intervention”, “Control intervention”; or “Don’t know”). If they answer, “Don’t know”, they are then asked to make a forced choice decision between active and control intervention [93]. Free text responses are collected regarding the reasons behind their choice (“What led you to believe that you received this treatment?”) and if they were unblinded (“If you discovered which treatment [active or control] you received, please tell us when and/or how you found out.”). Treating physiotherapists are also asked at trial cessation whether they thought they were in the active or control intervention group using similar methodology (three options including “Don’t know”, followed by forced choice and free text responses).

#### Therapist related factors

Therapist’s beliefs about exercise and about pain will be assessed via the modified Therapist Beliefs about Exercise Questionnaire [94] and the modified Pain Attitudes and Beliefs Scale for Physiotherapists (PABS-PT) [95], respectively. Therapist’s pain knowledge will be assessed using the revised Neurophysiology of Pain Questionnaire (rNPQ) [96] in the EPIPHA-KNEE group only to preserve therapist blinding in the Control group. Therapist’s perceptions of intervention credibility will be assessed using the Modified Intervention Credibility and Expectancy Questionnaire [87]. Last, therapist’s perceived connection with the participant will be self-rated using a 100 mm VAS Connectedness Scale (0 = no connection at all; 100 = maximum connection).

### Trial sample size

The Osteoarthritis Research Society International (OARSI) recommends an effect size (d = mean difference/SD_difference_) of 0.5 as the minimal clinically important difference (MCID) for outcomes in OA trials [97]. Past work has estimated that PSE increases physical activity by 1165 steps/day (SD = 985) in people with back pain, producing a large effect (d = 1.18) [39]. Given ~ 850 steps/day increase in knee OA populations after intervention (SD_difference_ = 950) [98] and our pilot data, we anticipate a moderate-large effect in the PSE group and a small effect in the control group at 12 months; consistent with a between group effect size of 0.5. Analysing these data using linear mixed effects modelling, to detect an effect size of d = 0.5 for physical activity using alpha = 0.05, power = 0.90, 4 time points, repeated measures correlation = 0.7, and 20% loss to follow-up [98], 156 participants (78/group) are needed. To additionally adjust for clustering based on therapist (~ 12 therapists/group; ~ 6.5 patients each; ICC = 0.05 [99]), 198 participants (99/group) are then required. The MCID for WOMAC Total at 12 months in people with knee OA undergoing an exercise intervention is 11.5 [100, 101], and given a between group SD from our pilot data of 13.8 (d = 0.83) [42], we are also sufficiently powered for this outcome. We did not adjust the alpha level for multiple comparisons (two primary outcome measures) in our power analysis, but we will present unadjusted *p*-values and confidence intervals for meaningful interpretation.

### Analysis

A full statistical analysis plan will be published prior to undertaking the formal analysis of collected data (including description of secondary analyses). The proposed preliminary analysis plan is detailed below.

#### Primary analysis

Data will be analysed using intention-to-treat principles with the trial biostatistician blinded to group allocation. We will use linear mixed effects models with random intercepts for individuals (to account for correlation for repeated measures) and will analyse the effect of treatment separately for each outcome. The model will include terms for stratification factors of sex and site. A time-varying, group specific effect (for each group) will be included as covariates for participants with restricted physical activity as a result of location-specific COVID-19 restrictions (based upon official activity restrictions and/or participant reported restrictions; assessment detailed above). Statistical significance will determine whether group-specific covariates or even any COVID-19 related effects are warranted for model parsimony. Multiple imputation will be used for missing data if the assumptions are reasonable [102]. We will construct linear contrasts to compare the adjusted mean change in outcome from baseline to each time point between the EPIPHA-KNEE group and the control group (and 95% confidence interval) to obtain estimates of the intervention effect. We will analyse primary and secondary outcomes without adjustment for multiple measures, but we will present unadjusted *p*-values and confidence intervals for all pre-specified analyses [103, 104] so readers can make adjustments (e.g., Bonferroni) at the level of Type I error control that they wish, at risk of making Type II errors.

#### Health economic analysis

A cost-utility analysis will be undertaken from the perspective of the health system. This will be published separately to the main trial findings. The main outcome measure will be quality adjusted life years (QALYs) gained in the intervention group over the 12 month follow-up period as compared with QALYs gained in the control group. To calculate the QALYs gained, the responses to the EQ-5D-5L will be converted into a utility score anchored on a scale from 0 (indicating a health state equivalent to being dead) and 1 (indicating full health) using available preference weights from an Australian general population sample [73]. These utility scores will be converted into QALYs gained for each individual participant by combining utility scores with the information about the time the participant spent experiencing that utility using area under the curve methods. We will also utilise the primary clinical outcome measures from the trial (WOMAC Total physical activity levels) as secondary outcome measures for assessing cost effectiveness. The incremental cost effectiveness ratios (ICER) comparing the costs for an improvement in the outcome (i.e. QALYs) in the intervention group as compared to the control group will be presented and their associated confidence intervals estimated. Cost effectiveness acceptability curves for varying threshold values of cost effectiveness will also be presented. An assessment of the sensitivity of the results obtained to variation in measured resource use effectiveness and/or unit costs will be undertaken using appropriate one-way and multi-way sensitivity analysis [105, 106]. A detailed health economic analysis plan will be published online prior to analysis and unblinding.

### Mediation analysis

We will use causal mediation analysis [107] to understand the mechanisms by which PSE affects physical activity and OA symptoms. We will deconstruct the treatment effect into indirect effects via changes in conceptualisation of pain/osteoarthritis, self-efficacy, implicit movement bias, implicit environmental bias, and pain sensitivity to activity.

### Ethics

The trial has ethical approval from the University of South Australia Human Research Ethics Committee (HREC No. 20237), the Central Adelaide Local Health Network Human Research Ethics Committee (HREC No. 12579), the University of Melbourne Human Research Ethics Committee (HREC No. 2057540), and Flinders University Human Research Ethics Committee (HREC No. 4478). All participants provide written informed consent for trial participation and for access to Medicare Benefits Scheme and Pharmacological Benefits Scheme data to allow cost effectiveness analysis.

## Discussion

Given the increasing burden of knee OA, there is an urgent need for new treatments that promote long-term adherence to physical activity in order to sustain its clinical and health benefits [28]. While evidence for effectiveness of PSE on altering unhelpful beliefs, and improving pain, function and movement/activity has been shown in musculoskeletal conditions such as back pain [29, 35–37, 39], it has never before been rigorously evaluated in the context of knee OA. Given that people with knee OA are typically provided with clear findings of structural joint changes, whereas those with back pain are typically not (i.e., ~ 90% of back pain is considered ‘non-specific’, with no structural cause [108]), there may be important differences in the degree to which information highlighting the contribution of central/cognitive factors to pain (versus peripheral, joint-based factors) is accepted. Further, given the established benefits of structured physical activity in reducing pain and disability in people with painful knee OA [3, 4, 8], there is great value in determining whether integrating PSE with a structured physical activity program can improve long-term adherence to activity and reduce OA symptoms.

This trial will be the first to investigate the clinical- and cost-effectiveness of integrating a physiotherapist-led contemporary PSE with an individualised walking, strengthening and general OA education program in knee OA, compared to that program alone. Findings from this study will inform health care providers about the benefit of incorporating PSE into recommended structured physical activity interventions for knee OA. Mediation analyses from this study will also allow us to evaluate the mechanisms by which the PSE intervention worked (or potentially, the reasons it did not), which will enable further improvement of the intervention by identifying potential mediators of therapeutic importance.

A potential limitation of this superiority trial relates to differing treatment duration between intervention groups. While the two groups have an identical number and timing of treatments, each in-person session is longer in the EPIPHA-KNEE group than the control group to accommodate the additional educational content. Such differences may make it difficult to determine whether it is the educational content itself, or the increased time with the therapist that underlies improved outcomes that may be seen. We considered using a sham intervention to increase the control group treatment time to match that of the EPIPHA-KNEE group. However, past work supports that sham educational options can be difficult to devise. For example, a recent trial evaluated the efficacy of PSE in acute low back pain, comparing to a ‘sham education’ control, and showed that sham education increased self-efficacy [109]. While such a sham intervention may capture non-specific features of the educational intervention that contribute to clinical outcome, it becomes problematic if the intervention of interest (PSE) may also work via a similar causal pathway (e.g., by increasing self-efficacy). Further, non-educational sham options may not be ideal. In our previous pilot study, we found that use of sham (deceptive) ultrasound was not well received by treating therapists and induced differing treatment expectations relating to physiotherapy between intervention groups (expectations met in control group versus violated in PSE group) [42].

In the absence of a sham component, it becomes most relevant to consider that a participant’s increased time with the treating therapist (e.g., in the EPIPHA-KNEE group receiving enhanced PSE) may foster stronger therapeutic alliance. However, it is important to note that strong therapeutic alliance not only has the potential for positive effects on clinical outcomes [110], but it may also have negative effects on some clinical outcomes (e.g., fear of movement [111]). Further, situations of poor therapeutic alliance or connectedness may also have negative influences on clinical outcome [112], negating an overall positive effect of increased therapist time on outcome. We will capture data on perceived connectedness in the therapeutic encounter, allowing us to formally explore such a possibility in our analyses. Last, the choice of long-term primary timepoints (12 months) also reduces the risk of increased therapist time influencing clinical outcome, given that a minimum of 3 months occurs between the last in-person session and the assessment timepoint of the primary outcomes.

The findings of this trial will provide Class I evidence for the clinical utility and cost-effectiveness of PSE in the management of symptomatic knee OA. The findings will provide evidence for the routine use of PSE-enhanced education and activity for people with knee OA in Australia. No similar studies have been completed to date in Australia or internationally, thus our findings will be of relevance at both the national and international level.

## Supplementary Information

**Additional file 1.**

## Data Availability

The datasets used and/or analysed during the current study will be available from the corresponding author (tasha.stanton@unisa.edu.au) on reasonable request once the study has been completed.

## Abbreviations

BMI: Body Mass Index
CEQ: Credibility/Expectancy Questionnaire
COVID-19: Coronavirus Disease 2019
EPIPHA-KNEE: Explaining Pain to Increase Physical Activity in people with Knee osteoarthritis
FREKAQ: Fremantle Knee Awareness Questionnaire
HREC: Human Research Ethics Committee
MBS: Medicare Benefits Scheme
MCID: Minimal Clinically Important Difference
NICE: National Institute for Health and Care Excellent
NRS: Numeric Rating Scale
OA: osteoarthritis
OACS: Knee OA and Activity Conceptualisation Scale
OARSI: Osteoarthritis Research Society International
PBS: Pharmacological Benefits Scheme
PCS: Pain Catastrophizing Scale
PABS-PT: Pain Attitudes and Beliefs Scale for Physiotherapists
PROMIS: Patient-Reported Outcomes Measurement Information System
PSE: Pain Science Education
PSEQ: Pain Self-Efficacy Questionnaire
QALY: Quality-Adjusted Life Year
RCT: Randomised Controlled Trial
REDCap: Research Electronic Database Capture
rNPQ: revised Neurophysiology of Pain Questionnaire
SA: South Australia
SD: Standard Deviation
SMART: Specific, Measurable, Achievable, Relevant, Time bound
SPIRIT: Standard Protocol Items: Recommendations for Interventional Trials
TIDieR: Template for Intervention Description and Replication
VAS: Visual Analogue Scale
WOMAC: Western Ontario McMaster
