# Supplementary material for: The EPIPHA-KNEE trial: Explaining Pain to target unhelpful pain beliefs to Increase PHysical Activity in KNEE osteoarthritis – a protocol for a multicentre, randomised controlled trial with clinical- and cost-effectiveness analysis
Source: BMC Musculoskelet Disord. 2021 Aug 28;22:738. doi: 10.1186/s12891-021-04561-6 (PMC8401372; doi:10.1186/s12891-021-04561-6)
Supplement: Supplementary file 1 — Additional file 1. [file 12891_2021_4561_MOESM1_ESM.pdf]

KNEE OA Trial

Exercise sheet 1

EXERCISE INSTRUCTIONS

1 Partial wall squats

Starting position

Gently lean against wall
Shoulders, back and buttocks resting against wall

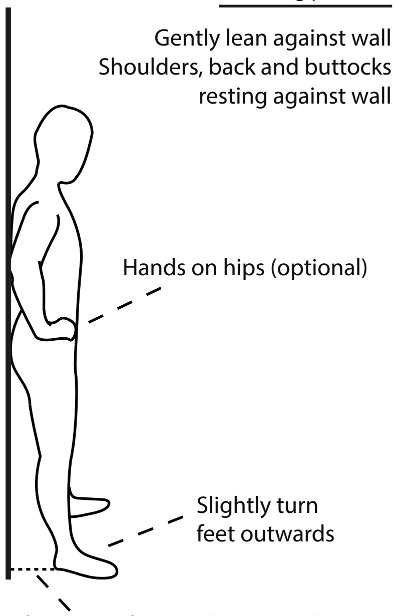

Exercise

Slowly slide down and back up wall
Stop before knees cover toes
(30° knee bend)

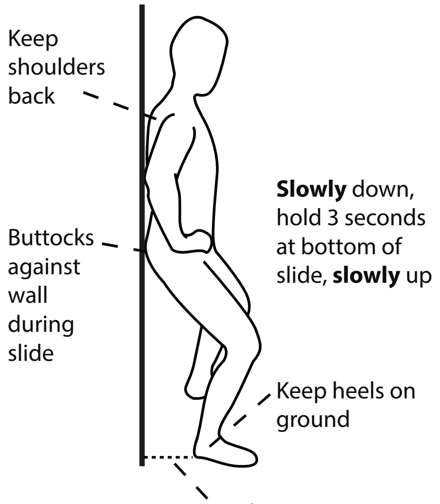

Variations of exercise

Hold for 5 seconds at bottom of slide

Move non-study leg further from wall

More weight on study leg by shifting body weight across

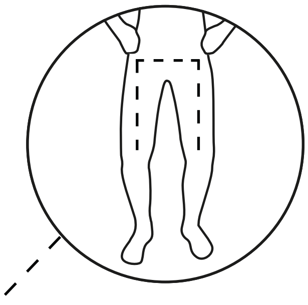

During squat:
Keep knees hip-width apart
Do not collapse knees inwards

2 Sliding

Starting position

Stand on study leg
Non-study leg on sliding surface

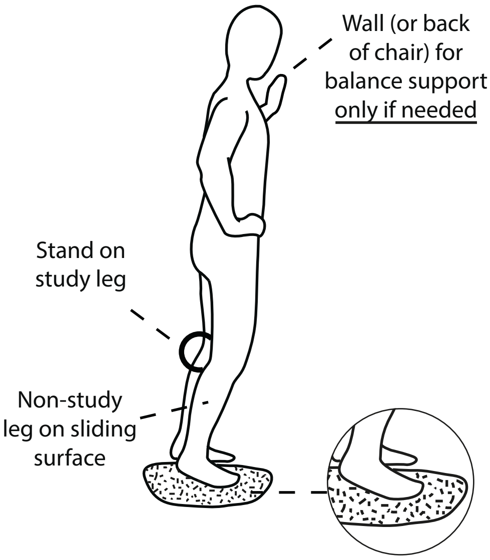

Smooth flooring: use towel under foot
Carpet: use plastic bag over foot

Exercise

Slowly slide forwards and backwards with non-study leg
Start with smaller slides (few inches)

If balance support needed, use wall (or back of chair)
Avoid leaning body weight onto arm

Variations of exercise

Progress by making larger slides as you gain control

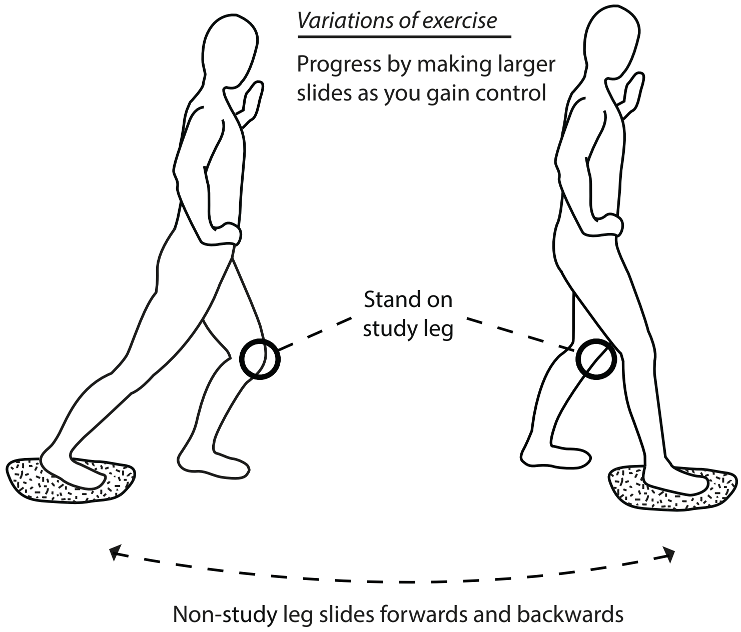

Thanks to our patients who reviewed and helped us test these exercise sheets.

Graphic Design by Lauren Bardin, 2020©

KNEE OA Trial

Exercise sheet 2

EXERCISE INSTRUCTIONS

3

Sit-to-stand (from chair)

Starting position

Sit on firm, stable chair

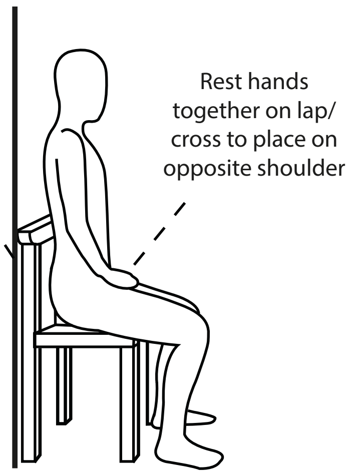

Exercise

Lean slightly forward
Move body weight forwards, nose over knees
Slowly up and **slowly** down

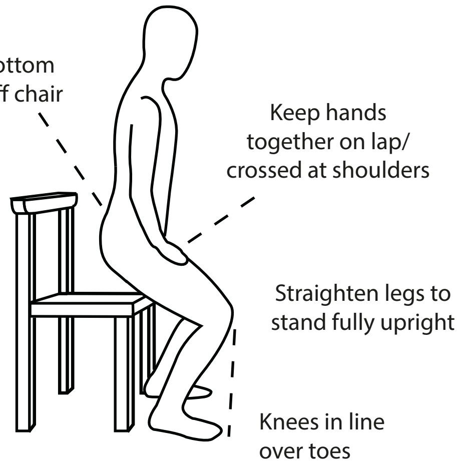

Variations of exercise

Hold 3 seconds before touching down

Lean body weight more over study knee

Move non-study knee further from chair

4

Stepping (forward/backwards)

Starting position

Stand on study leg
If using balance support, avoid leaning body weight onto arm

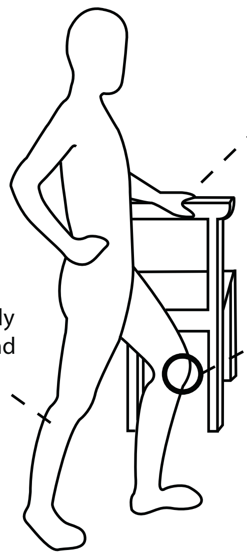

Exercise

Slowly step non-study leg forward to touch floor in front
Step backwards again to starting position

Keep study leg slightly bent with weight on study leg throughout the exercise

Variations of exercise

Progress by taking larger steps as you gain control

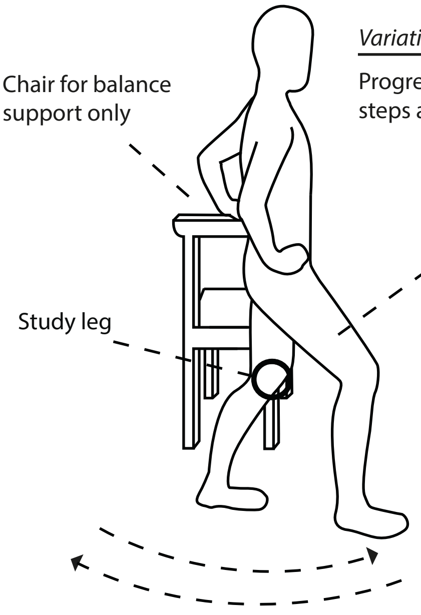

Thanks to our patients who reviewed and helped us test these exercise sheets.

Graphic Design by Lauren Bardin, 2020©

KNEE OA Trial

Exercise sheet 3

EXERCISE INSTRUCTIONS

5 Step Ups

Starting position

Stand with study leg on a step
If no steps, use phone books

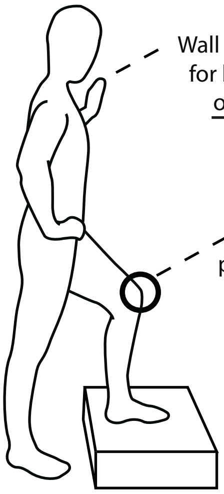

Wall (or back of chair) for balance support only if needed

Study knee pointing forward and positioned over foot

Standard: low step
Advanced: higher step

Exercise

Step up slowly taking body weight through study leg (don't push off with foot of non-study leg)

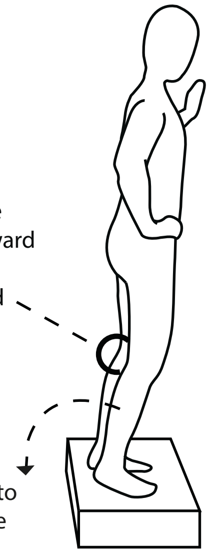

Study leg, keep knee pointing forward and positioned over foot

Wall (or back of chair) for balance support only

Non-study leg steps back to starting position to complete exercise

Variations of exercise

Progress to increased step height

6 Step Downs (forward touch downs)

Starting position

Stand on step facing downstairs

Wall (or back of chair) for balance support only if needed

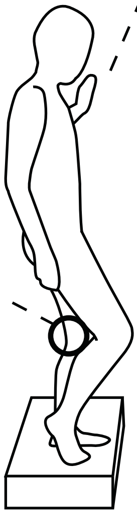

Study knee pointing forward

Standard: low step
Advanced: higher step

Exercise

Bend study knee slowly and **lower non-study leg** towards the ground

Keep study knee pointing forward during the movement

Straighten study knee slowly to return to starting position

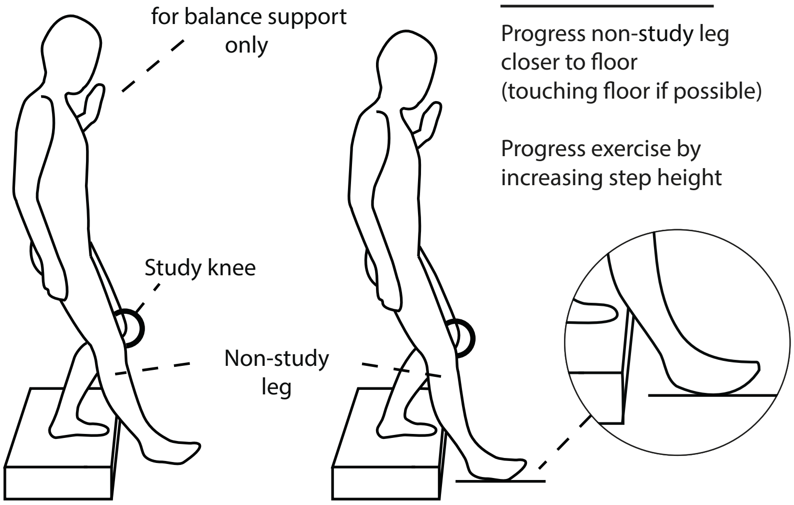

Wall (or back of chair) for balance support only

Study knee

Non-study leg

Variations of exercise

Progress non-study leg closer to floor (touching floor if possible)

Progress exercise by increasing step height

Thanks to our patients who reviewed and helped us test these exercise sheets.

Graphic Design by Lauren Bardin, 2020©
